# Building on Existing Classifications of Behavior Change Techniques to Classify Planned Coping Strategies: Physical Activity Diary Study

**DOI:** 10.2196/50573

**Published:** 2023-12-18

**Authors:** Maya Braun, Helene Schroé, Annick L De Paepe, Geert Crombez

**Affiliations:** 1 Department of Experimental-Clinical and Health Psychology Ghent University Gent Belgium; 2 Department of Movement and Sports Sciences Ghent University Ghent Belgium

**Keywords:** behavior change, problem-solving, classification, personalization, physically active, digital intervention, solution, techniques, plan, physical activity

## Abstract

**Background:**

When trying to be more physically active, preparing for possible barriers by considering potential coping strategies increases the likelihood of plan enactment. Digital interventions can support this process by providing personalized recommendations for coping strategies, but this requires that possible coping strategies are identified and classified. Existing classification systems of behavior change, such as the compendium of self-enactable techniques, may be reused to classify coping strategies in the context of physical activity (PA) coping planning.

**Objective:**

This study investigated whether coping strategies created by a student population to overcome barriers to be physically active can be mapped onto the compendium of self-enactable techniques and which adaptations or additions to the frameworks are needed.

**Methods:**

In total, 359 Flemish university students created action and coping plans for PA for 8 consecutive days in 2020, resulting in 5252 coping plans. A codebook was developed iteratively using the compendium of self-enactable techniques as a starting point to code coping strategies. Additional codes were added to the codebook iteratively. Interrater reliability was calculated, and descriptive statistics were provided for the coping strategies.

**Results:**

Interrater reliability was moderate (Cohen κ=0.72) for the coded coping strategies. Existing self-enactable techniques covered 64.6% (3393/5252) of the coded coping strategies, and added coping strategies covered 28.52% (n=1498). The remaining coping strategies could not be coded as entries were too vague or contained no coping strategy. The added classes covered multiple ways of adapting the original action plan, managing one’s time, ensuring the availability of required material, and doing the activity with someone else. When exploring the data further, we found that almost half (n=2371, 45.1%) of the coping strategies coded focused on contextual factors.

**Conclusions:**

The study’s objective was to categorize PA coping strategies. The compendium of self-enactable techniques addressed almost two-thirds (3393/5252, 64.6%) of these strategies, serving as valuable starting points for classification. In total, 9 additional strategies were integrated into the self-enactable techniques, which are largely absent in other existing classification systems. These new techniques can be seen as further refinements of “problem-solving” or “coping planning.” Due to data constraints stemming from the COVID-19 pandemic and the study’s focus on a healthy Flemish student population, it is anticipated that more coping strategies would apply under normal conditions, in the general population, and among clinical groups. Future research should expand to diverse populations and establish connections between coping strategies and PA barriers, with ontologies recommended for this purpose. This study is a first step in classifying the content of coping strategies for PA. We believe this is an important and necessary step toward digital health interventions that incorporate personalized suggestions for PA coping plans.

## Introduction

### Background

Engaging in physical activity (PA) may decrease all-cause mortality and risk of several chronic conditions [1]. The World Health Organization [2] recommends that healthy adults are moderately and physically active for 150-300 minutes per week, vigorously and physically active for 75-150 minutes per week, or an equivalent combination of both [2]. However, about 27.5% worldwide do not meet those guidelines, rising to 36.8% in high-income Western countries [3]. To promote PA, it is important to understand why individuals are physically inactive. There are several theoretical models that can help us in answering this question [4].

Social cognitive models of health behavior describe intention as the most proximal and powerful predictor for behavior, which is why they focus on antecedents of intention such as attitude, subjective norm, and perceived behavioral control [5,6]. However, largely neglected in these models is the gap between intention and behavior. A meta-analysis reported that 46% of people who intended to be more physically active were not able to successfully do so [7]. The health action process approach [8] addresses this intention-behavior gap by including additional self-regulation components. The model proposes that individuals who have an intention to engage in a specific behavior can use techniques such as planning the behavior (known as action planning), monitoring progress, identifying potential obstacles to behavior change, and developing effective coping strategies to overcome them (known as coping planning or problem-solving strategies). By including these additional processes, the health action process approach model provides a more comprehensive approach to understanding and promoting behavior change.

The effect of action and coping planning in bridging the PA intention-behavior gap has been corroborated in multiple studies [9-15]. Further, studies have investigated how, when, and for whom action and coping planning works. Examples of individual characteristics that influence how effective planning is are age [11], impulsivity [16], self-efficacy [17], and motivational stage [13,18,19]. When it comes to plan characteristics, some studies have found that instrumentality and high specificity are important for behavior change [20,21]. Others found that formulating the plan around a specific contextual cue, such as starting an activity after another one was finished, while allowing flexibility in other plan aspects is beneficial [22,23].

Although research about for whom, how, and when planning works is valuable, the *content* of relevant plans may also vary substantially depending on the type of barrier, the type of planned activity, and various individual and contextual characteristics. For example, someone with a flexible working schedule might easily adapt a plan to a different time of the day, but this might not be possible for someone with fixed working hours and other responsibilities. Similarly, even though coping strategies that are focused on an individual’s motivation might be relevant for activities that are enacted alone, they might be less appropriate for group activities.

A possibility to personalize plans is to rely on the expertise of the individuals, who know themselves and their context best. This has been done in several digital health interventions. For example, in a study by Degroote et al [24], participants had to formulate action and coping plans for every day of the study. However, individuals found that creating plans from scratch was too effortful [24], and the quality of self-formulated plans was often low [25]. This was especially true for coming up with coping strategies in the context of coping plans [24,25]. Participants explicitly wished for more support in creating coping plans, for example, in the form of relevant suggestions [24]. Digital interventions can provide such support in generating coping plans, which would reduce user effort and allow plans of high quality. However, determining which plans are relevant for which user under which circumstances requires a variety of information, such as information on the user, their action plan, and barrier as well as the broader context. In the first step, a comprehensive framework of the possible content of these plans is crucial for effective implementation. Currently, there is no framework available that captures the content of coping strategies. However, we might be able to reuse existing frameworks from other contexts.

In its essence, the coping strategy within a coping plan is a strategy to facilitate behavior change despite existing barriers. As such, it is an implementation of a behavior change technique (BCT). A BCT is defined as “an observable, replicable, and irreducible component of an intervention designed to alter or redirect causal processes that regulate behavior” [26]. It is considered the smallest active ingredient in a behavior change intervention. As such, BCTs should be suitable to describe coping strategies. For example, “adding post-its to your bathroom mirror” to remind you to take the bicycle to work instead of the car corresponds to the BCT “prompts or cues,” while “looking up how to carry out specific yoga poses” corresponds to “instruction on how to perform the behavior.” However, not all BCTs will be suitable to be used in the context of coping strategies. For example, while self-reward is a potential coping strategy that a person can implement by themselves, other forms of rewards are not because they require other people to do the rewarding. The most well-known framework to categorize BCTs is the BCT taxonomy by Michie et al [27], but similar frameworks have been developed in order to capture techniques used in interventions [28-33]. Even though taxonomies, and classification systems in a broader sense, are crucial for research, it is unclear whether either of those will be sufficient when it comes to describing an individual’s coping strategies due to multiple reasons.

First, with the increased focus on self-management and empowerment [34], there is a need for a comprehensive classification system that considers coping strategies that are created and enacted by the individual themselves, as opposed to by someone or something delivering an intervention. Similar considerations have prompted Knittle et al [35] to develop a compendium of self-enactable techniques. The compendium contains 123 techniques that individuals can carry out themselves in order to achieve behavioral change and maintenance.

Second, the level of specificity varies among BCTs. While some techniques are highly specific, such as “information about antecedents” or “prompts or cues,” other techniques are less specific. For example, the BCT “problem-solving” is defined as “Analyse, or prompt the person to analyse factors influencing the behavior and generate or select strategies that include overcoming barriers and/or increasing facilitators” within the BCT taxonomy. Problem-solving is a complex and multifaceted construct and may consist of various other techniques. To achieve personalized eHealth interventions, it may be necessary to further specify some techniques such as problem-solving.

### This Study

This study investigated whether the coping strategies created in the context of problem-solving for PA plans can be conceptualized using existing taxonomies, such as the compendium of self-enactable techniques by Knittle et al [35] as a starting point. To do this, we instructed a sample of students to create coping plans for PA, as reported for 8 consecutive days in a morning diary by psychology students. The first aim was to investigate how many and which coping strategies were (1) fully covered by existing classifications of techniques, (2) covered but not in sufficient detail, or (3) not covered. The second aim was to propose which adaptations or additions would be necessary in order to fully capture those coping strategies.

## Methods

### Description of the Data Set

#### Participants

Participants in this study were students in the first year of the master’s degree in clinical psychology at Ghent University, Belgium. The data were part of an educational task of the course “Models in Health Psychology,” and students provided informed consent for the use of their data for research purposes.

In total, 377 (99.2%) of the 380 students provided informed consent, of which 359 (n=318, 88.6% female) provided valid data. Reasons for exclusion are detailed in the *Data Processing and Analysis* section. Age ranged from 19 to 48 years with a mean of 20.94 (SD 3.18) years and a median of 20 (IQR 20-21) years.

#### Ethical Considerations

This study was conducted from October 2020 until November 2020 and was approved by the ethical committee from the Faculty of Psychology and Educational Sciences at Ghent University (registration 2020/87). Participants provided consent for data analysis before completing the intake questionnaire. Those who did not consent still completed all questionnaires for educational purposes but were excluded from analysis, and their results were not saved beyond the scope of the assignment. All data have been pseudonymized by removing all names, email addresses, student IDs, and references to specific locations from the data set. Students did not receive any compensation for agreeing to share their data for research purposes, as they already had to complete the questionnaires as part of an assignment.

#### Procedure and Material

Data were collected in a web-based task called “MyActionPlan” using the web-based survey software LimeSurvey (LimeSurvey Project). The web-based task consisted of 3 different questionnaires to collect data from participants: an intake questionnaire, a morning questionnaire, and an evening questionnaire. An overview of the full task was uploaded to the Open Science Framework (OSF) [36]. The questionnaires were tested by friendly users.

The intake questionnaire had 5 parts. The first part collected demographic data. The second part had 30 statements on PA determinants, such as motivation and self-efficacy. The third part explored the living environment using items from the Neighborhood Environment Walkability Scale [37]. In the fourth part, participants reported PA from the previous week based on the International Physical Activity Questionnaire—Short Form [38] and received feedback on meeting the World Health Organization’s PA recommendations. Finally, the fifth part asked participants about disliked activities. This questionnaire contained 39 questions on 9 pages.

After completing this initial questionnaire, participants received a summary and were reminded that they had to complete questionnaires in the morning and evening of the coming 8 days. Participants did not receive any further prompts to fill in the morning and evening questionnaires.

The morning questionnaire consisted of various components. In the first part, participants were instructed to set a PA goal in minutes or steps and report any changes they made to their goal compared to the previous day, along with the reasons for such alterations. In the second part, they created up to 3 action plans, with each plan accompanied by up to 2 coping plans. For this, they were prompted to think about each part of their plan in separate questions (ie, “What are you going to do?” “When are you going to do it?” “Where will you do it?” and “Who are you going to be active with?” for the action plan and “What is the most important barrier for achieving your activity goal?” and “How can you overcome this barrier?” for the coping plan), which they were asked to answer in open-text format. They were also asked to summarize their action plan in 1 sentence.

In the third part, participants provided some information about current physical and emotional states on a Likert scale from 1 to 7 as well as the expected weather and the expected business of their day. Upon the completion of the morning questionnaire, participants received a summary of their actions and coping plans via email. This questionnaire contains 26 questions for participants who created 1 action plan with 1 coping plan, 5 additional questions for each additional action plan, and 2 additional questions for each additional coping plan. Questions are presented on 6 separate pages, with 1 additional page for each additional action plan and each additional coping plan.

The evening questionnaire was completed at the end of each day for 8 consecutive days. Participants were asked to rate the degree of success of their PA goal on a scale of 1-5 and reflect on the reasons underlying their success or failure. They were also asked to provide information about BCTs they have applied to achieve their PA goal that they had not reported yet. After completing the evening questionnaire, participants received an email with a summary of that day’s evaluation of their PA goal. The questionnaire contains 4 questions that are presented on 2 pages.

Completeness checks were added to all questionnaires, not allowing participants to proceed without completing all questions. Incomplete questionnaires were not used in data collection. Participants were able to review their answers and return to them at any point before submitting. Each participant had a unique token in order to connect the results from different questionnaires.

### Data Coding and Analysis

#### Creating the Coding Scheme

Action and coping plans created within the morning questionnaire were coded into different categories in order to facilitate further analyses. Action plans were coded into activity (“What are you going to do?”), time (“When are you going to do it?”), location (“Where are you going to do it?”), and social context (“With whom are you going to do it?”). Coping plans were coded into barriers and coping strategies. A research team of 6 researchers (the researchers MB, HS, and 4 master’s thesis students—2 from clinical psychology and 2 from health and movement sciences) iteratively developed the codebook. The coding of the coping strategies will be discussed in more detail.

First, MB and HS developed an initial codebook. For the coping strategies, the categories were based on the self-enactable techniques by Knittle et al [35]. The choice for the self-enactable techniques over the more commonly used BCTs [27] was made as there is a significant overlap between the 2 systems, with the perspective of the self-enactable techniques matching self-developed plans more closely.

Commonly occurring coping strategies were placed in a separate file with examples from the data in order to facilitate coding, but all self-enactable techniques could be used for coding. The codebook was improved in 3 rounds. In each round, all researchers independently coded the same approximately 50 action and coping plans. MB and HS then compared the results and summarized them. These results were the base of a discussion among the research team, which led to improvements to the codebook. All researchers were encouraged to provide feedback throughout the coding process and add examples to categories. The initial and final versions of the codebook can be found on OSF [36].

#### Coding

The coding of the coping strategies based on the codebook was divided between 4 master’s thesis students and was performed in Microsoft Excel (Microsoft Corp). Of the 2896 reported days, 396 (13.7%) were independently double-coded. This resulted in 680 (14%) of 4843 coping strategies being double-coded, as participants could provide multiple action plans per day and multiple coping plans for each action plan.

#### Data Processing and Analysis

All data processing and analysis were conducted in RStudio (R Foundation for Statistical Computing). First, the data sets from intake, morning, and evening questionnaires were merged. Second, descriptive statistics were used to provide sociodemographic information of the sample. The resulting data set can be found on OSF as well as the analyses carried out on it [36]. Third, in order to describe the content of the coping strategies, frequency statistics of the coping strategies were calculated based on the coding provided by the primary rater of all formulated coping strategies. Fourth, in order to measure interrater reliability, the unweighted Cohen κ [39] was calculated between rater A and rater B using the *κ2* function from the *irr* package [40]. Only plans that both rater A and rater B considered valid coping strategies were included in this calculation.

## Results

### Present Codebook

The final version of the codebook consists of 3 kinds of codes. First, codes that stem directly from the compendium of self-enactable techniques were included. Second, codes were added by the researchers in order to code coping strategies that were either not mentioned or insufficiently specified within the existing classification system. Third, codes for input that were too unspecific in terms of behavior change (eg, “motivate myself”), contained multiple coping strategies with no clear prioritization (eg, “I will put on weatherproof clothes, or exercise indoors instead”), or contained no coping strategy at all (eg, empty fields, “I will just do it”) were added.

An overview of the added codes for techniques and their definitions can be found in Table 1. In the following paragraphs, the reasoning for the additions is provided along with examples from our study. First, a class of techniques “plan adaptation” was added, with a further detailing of the part of the plan that will be changed. Specifically, the techniques are “plan adaptation (other activity),” “plan adaptation (other moment),” “plan adaptation (other location),” and “plan adaptation (shorter duration).” This was considered an additional class of techniques because it does not ensure that the original plan is carried out. Instead, it aims for a change of the planned target behavior in case of barriers. This might mean that the target behavior is carried out to a lesser extent than originally planned or takes a different form. This was found to be an important addition. Many techniques within the compendium of self-enactable techniques focus on internal processes, such as motivation, knowledge, or beliefs. In contrast, the reported barriers are often (perceived) external barriers, such as (perceived) lack of time or (perceived) lack of adequate space to perform the activity.

**Table 1:** Overview of all added techniques.

Name	Definition
Plan adaptation	A self-enactable technique where one changes the original action plan in order to manage practical barriers
Plan adaptation (other activity)	A plan adaptation technique concerning the type of activity one carries out
Plan adaptation (other moment)	A plan adaptation technique concerning the moment when one carries out the activity
Plan adaptation (other location)	A plan adaptation technique concerning the location where one carries out the activity
Plan adaptation (other company)	A plan adaptation technique concerning with whom one carries out the activity
Plan adaptation (other duration)	A plan adaptation technique concerning the duration of the planned activity.
Time management	A self-enactable technique where one first manages other responsibilities and plans unrelated to the planned activity in order to prevent interference with the plan
Manage negative bodily states	A self-enactable technique where one takes steps to reduce negative physical states, such as fatigue, hunger, thirst, or pain, to facilitate the performance of the target behavior
Ensure availability of material	A self-enactable technique where one ensures the presence and functioning of any required material for a planned activity
Do it together	A self-enactable technique where one carries out the planned activity together with others

Second, the technique “time management” was added. This refers to planning out parts of the day that ensure that nothing interferes with the action plan. Examples of this could be making sure to get household chores done in the morning so that one could do yoga in the evening or to make sure to leave work on time to be able to join an exercise class.

Third, “manage negative bodily states” was added. This is equivalent to the technique “manage negative emotions,” focusing on bodily states instead of emotional ones. It refers to taking steps to reduce negative bodily states, such as fatigue, hunger, thirst, or pain, to facilitate the performance of the target behavior. Within our sample, this often covered eating or drinking at appropriate moments, taking a nap or ensuring one gets enough sleep, or stretching before exercising.

Fourth, we added “ensure availability of material.” This refers to ensuring that required materials for a planned activity are available and functioning. For example, participants would make sure that their bicycles are working properly if they had not used them in a while. This technique does not describe preparing materials by, for instance, packing a bag for the gym.

Finally, we added the category “do it together” to the codebook. Participants often reported they would ask another person to join their activity. This action leaves open whether others are asked to join as emotional support, to make the activity more pleasant (task crafting enjoyment), or to integrate the goal of exercise with the goal of socializing (goal integration).

### Interrater Reliability

Coping strategies that could not be coded were excluded from the analysis (n=46). Unweighted Cohen κ for the double-coded coping strategies (n=661) is 0.72, which is commonly interpreted as moderate agreement [41]. Upon closer investigation of differently coded items, a majority concerned 1 researcher coding 1 technique and another researcher coding multiple techniques. Further, some differences were found between existing techniques that can be similar in practice, such as public commitment and social support. Few differences were found between coders when it came to the techniques added within this study. Due to sufficient interrater reliability and the nature of the differences on the one hand, and as agreement between coders was not deemed feasible as a method of dealing with interrater differences within this study due to the volume of data, further analyses were carried out based on the coding created by the first coder.

### Descriptive Statistics

The absolute and relative frequencies of the different techniques are listed in Table 2. Techniques from the original compendium of self-enactable techniques covered 64.6% (n=3393/5252) of the coded coping strategies, whereas techniques that were added by the research team covered 28.52% (n=1498) of the coping strategies. The remaining 6.87% (n=361) of coping strategies were not specified as they were not clear or too unspecific or they provided no coping strategy at all.

**Table 2:** Frequencies of the techniques within our data set (N=5252).

Coping strategy			Frequency, n (%)
**Coping strategies from the compendium of self-enactable techniques (n=3393, 64.6%)**			
	Adding objects to the environment	1174 (22.35)	
	Prompts or cues	358 (6.82)	
	Public commitment	309 (5.88)	
	Reflect on reasons to perform the behavior	193 (3.67)	
	Task crafting (enjoyment)	173 (3.29)	
	Obtain emotional social support	163 (3.1)	
	Task crafting (skills and ability)	154 (2.93)	
	Self-incentive	152 (2.89)	
	Action control (maximize effort)	120 (2.28)	
	Action planning	114 (2.17)	
	Problem-solving	96 (1.83)	
	Verbal self-persuasion about own capability	61 (1.16)	
	Pharmacological support	59 (1.12)	
	Self-commitment	52 (0.99)	
	Goal integration	48 (0.91)	
	Obtain practical social support	32 (0.61)	
	Avoid cues for unwanted behavior	31 (0.59)	
	Reframing perspective on behavior	31 (0.59)	
	Observe demonstration of the behavior	19 (0.36)	
	Obtain information about health consequences	12 (0.23)	
	Self-monitoring of behavior	12 (0.23)	
	Conserve mental resources	11 (0.21)	
	Imaginary reward	6 (0.11)	
	Obtain instruction on how to perform the behavior	4 (0.08)	
	Behavior substitution	3 (0.06)	
	Reminder of outcome goal content	3 (0.06)	
	Social reward	2 (0.04)	
	Anticipated regret	1 (0.02)	
**Coping strategies that were added by the researchers (n=1498, 28.52%)**			
	Time management	472 (8.99)	
	Plan adaptation (other time)	231 (4.4)	
	Do it together	217 (4.13)	
	Manage negative bodily state	180 (3.43)	
	Plan adaptation (other activity)	175 (3.33)	
	Plan adaptation (other location)	125 (2.38)	
	Plan adaptation (other company)	50 (0.95)	
	Ensure availability material	44 (0.84)	
	Plan adaptation (other duration)	4 (0.08)	
**Coping strategies that could not be coded or answers that did not provide a coping strategy (n=361, 6.87%)**			
	Unclear coping strategy	195 (3.71)	
	No coping strategy	166 (3.16)	

## Discussion

### Principal Findings

This study investigated how coping strategies of PA coping plans can be categorized into existing categorization systems, using the compendium of self-enactable techniques as a start point. First, 64.6% (3393/5252) of the coping strategies could be coded using this compendium. Second, we identified 6 additional techniques, accounting for about 28.52% (n=1498) of the coded coping strategies. Third, some coping strategies (n=361, 6.9%) could not be coded as they were too vague or contained no coping strategy at all. Fourth, interrater reliability was moderate for the coded coping strategies.

Our findings indicate that existing categorization systems, such as the compendium of self-enactable techniques by Knittle et al [35] or the BCT taxonomy by Michie et al [27], are a good starting point to capture coping strategies for PA plans at the individual level but are currently insufficient to fully capture all strategies. Within this paper, we provide 9 potential techniques that could be added to an existing classification system. The majority of the techniques that were added mostly concerned adapting the existing plan in some ways, namely, by adapting the activity, location, company, starting time, or duration of the activity. While most coping strategies created in the context of problem-solving aim the individual to still perform the original plan (eg, go for a 15-minute walk at noon with a colleague), plan adaptation coping strategies allow the original plan to change, though some variation of the target behavior would still be performed (ie, PA). Furthermore, we added the technique “time management,” which is about facilitating the performance of the plan by scheduling around the activity, for example, by making sure that other chores are completed before the plan, and the technique “ensure availability of material,” which is about controlling ahead of a planned activity whether the required material is available and functioning.

There is room for discussion in how far the techniques added above are elaborations or specifications of existing BCTs, such as problem-solving, and whether they are only relevant for our use case—namely, planning for PA—or whether they are more generally applicable. The distinction between what is an example of a BCT as opposed to what is a BCT in itself is currently not clear. For example, practical social support is a kind of social support within the BCT taxonomy. However, it could also be interpreted as an example of what social support can look like. Similarly, “providing information about health consequences” could be further specified into “providing information about health risks” and “providing information about health benefits of performing a behavior,” which might influence behavior differently and have differential effects depending on person and behavior characteristics. On the other hand, these could just be 2 different implementations of “Information about health consequences.” The definition of BCTs as “irreducible components” [27] or more recently “smallest part of a behavior change intervention” [42] further opens the question whether all current BCTs are appropriately defined and are indeed *irreducible* or *the smallest part*. To date, there is no clear guidance beyond the definition of BCT on how to determine whether a specified technique is indeed a BCT or not. Recent developments concerning this will be discussed in more detail in the *Implications for Future Research* section.

While most self-enactable techniques focus on changing the individual and their behavior, such as skills, motivation, or self-monitoring, the added techniques focus on adapting one’s environment. Only few existing techniques consider the environment, such as “adding objects to the environment,” which accounts for 22.35% (n=1174) of coping strategies. It is unsurprising that more coping strategies focus on environmental factors (n=2371, 45.1% of all coping strategies coded when adding problem-solving, plan adaptation, time management, ensure availability of material, and adding objects to the environment) than on increasing motivation, given existing research on the intention-behavior gap [7], which suggests that 46% of behavior cannot be explained by factors related to intention alone.

We also added the class “manage negative bodily states” as a physical equivalent of “manage negative emotions.” While this class already accounted for 3.43% (n=180) of coded coping strategies in our sample of mostly young and healthy participants, we expect this to be all the more relevant for samples that are older or are dealing with chronic illness or other physical conditions. This class could also be relevant for performing PA at a sports level where physical preparation may be more necessary as more strain is being put on the body. The intervention mapping taxonomy [28] contains a similar technique called “improving physical and emotional states” that contains both the original technique from the compendium of self-enactable techniques “manage negative emotions” and the new suggestion of “manage negative bodily states.” Because techniques for managing emotions differ substantially from techniques for managing bodily states, we would propose having the 2 techniques apart—at least for the case of PA.

Finally, we added the class “do it together.” This class described any coping strategies where participants reported solving a barrier by performing the activity together with at least one other person. It should be noted that this class was merely useful for coding social coping strategies because participants did not provide information on why they wanted to add another person to an activity. However, it is not necessarily suitable for recommendations, as the attributes and links of such a coping strategy depend on its function. Social coping strategies of this kind could then be conceptualized as task crafting (enjoyment), goal integration, or any kind of social support within the compendium of self-enactable techniques [35].

### Implications for Future Research

We have argued for a content-level classification of coping strategies for PA behaviors. Such a classification should take into account different kinds of coping strategies, including those targeting contextual factors. Digital interventions could then depart from this classification in order to provide personalized suggestions for coping plans. For example, barriers surrounding a lack of time and their corresponding coping strategies can be suggested primarily on days that are busier based on integration with calendar applications or based on previous data from the user. Providing these personalized suggestions may reduce user burden [24] and improve the quality of plans. Similarly, coping strategies that recommend to shorten or divide activities may only be suggested for activities that have a minimum duration (eg, 30 minutes) and that can be shortened easily—we can recommend to someone to go on two 15-minute walk instead of one 30-minute walk, but two 15-minute swims are much less likely to be feasible.

To inform personalized recommendations for problem-solving, coping strategies will also need to be linked to relevant barriers. Future studies could evaluate the link between barriers and coping strategies in the evening questionnaire by inquiring which coping strategies worked when. Similar research has been done in the context of the Human Behaviour Change Project, linking BCTs to mechanisms of action in the Theory and Techniques Tool [43]. The Theory and Techniques Tool determines the link between BCTs and mechanisms of action using data from 2 studies, a literature synthesis study and an expert consensus study [43,44].

Beyond linking coping strategies to barriers based on data, domain experts should be involved in creating recommendations, as some links could be found in observational data that might not be appropriate to recommend (eg, participants withholding themselves food unless they go for a run) or could have negative effects on other health behaviors (eg, participants rewarding themselves with candy completing their goal).

To create links between barriers and coping strategies, it can be interesting to define specific attributes for each coping strategy. This allows researchers to create rules based on these attributes. A relatively simple rule could be that coping strategies with the property “focus on environment” cannot be used to solve internal barriers such as motivation. This was done to a small extent in the compendium for self-enactable techniques by adding notes and defining for each technique whether it requires external input. It also defines prerequisites where necessary. Other relevant attributes could be whether the coping strategy is short- or long-term, whether it requires specific knowledge, experience, or materials, and whether it is motivational or practical.

A system that would allow these kinds of relationships being built clearly and consistently could be created using ontologies [45]. Ontologies are computer-readable classification systems that are easily reusable and adaptable [46]. Within an ontology, each concept (class) is uniquely identified and unambiguously defined. The relationships between concepts can also be defined, allowing for hierarchical relationships (ie, “plan adaptation” is a kind of “problem-solving”), but also other kinds of relationships (ie, “plan adaptation” is a relevant coping strategy for “no time”). Moreover, each class can have attributes that further define it (eg, “plan adaptation” is a short-term coping strategy and “social support” requires another person to be involved). Rules can be created based on class properties that can automatically make connections between coping strategies and barriers [46].

However, building ontologies is time and resource intensive. That is why the reuse of existing ontologies is encouraged whenever possible. A recent preprint describes the development of an ontology of BCTs within the Human Behaviour Change Project [26]. This ontology is built upon the BCT taxonomy [27] by taking into account extensive user and expert feedback provided in a variety of formats and relevant research reports and other classification systems [47], including the compendium of self-enactable techniques [35] used within this study. While those do not have a sufficient level of granularity to fully map coping strategies in the context of PA coping plans, future work could build upon those ontologies by adding relevant coping strategies either as additional BCTs or as further specifications or implementations of existing BCTs. As ontologies are meant to represent knowledge for particular purposes in a given domain, collaboration and building upon each other’s work is crucial. Furthermore, ontologies are considered never to be finished and should be continuously updated based on the changing knowledge in the field. As such, the coding of PA plan coping strategies allowed us to provide feedback to the BCT part of the Behavior Change Intervention Ontology both within a structured evaluation and in informal discussion [26]. Given that context, we regard this paper as one of the first steps within a collaborative effort to formalize existing knowledge on health behavior change using ontologies.

### Limitations

This study has a number of limitations. First, as the plans were created by a young and healthy sample, some coping strategies that could be relevant to the general population may not have been mentioned (eg, coping strategies that required significant financial means). Future research should expand upon this study by using samples more representative of the general population or other target groups such as older adults or clinical populations. As different populations might face different barriers, additional coping strategies might be relevant. Second, data were collected in October and November 2020. During this time, measurements were in place to reduce the spread of the COVID-19 pandemic, limiting social interaction. Classes and clubs related to PA were largely closed, influencing the kinds of physical activities participants performed. This in turn influenced what kinds of barriers were faced and what kind of coping strategies were relevant. In a time period without government measures, we would expect an increased use of PA facilities, such as gyms, and an increased rate of activities in group. Barriers and coping strategies would then also reflect those changes. Third, codes by the first rater were used for descriptive statistics within this study, as intercoder agreement was not feasible due to the amount of data coded. As differences rarely concerned the newly created classes and interrater reliability was moderate, we do not expect this to influence the conclusions taken from this research. However, a careful review of intercoder differences is required before using these data for deeper analyses, such as connecting barriers to coping strategies. Finally, we only investigated which coping strategies were created and not which ones were most carried out or otherwise most appropriate. Further research needs to also take into account (1) whether the planned coping strategies were actually performed and (2) whether they resulted in increased PA behavior.

### Conclusions

This study explored the content of coping strategies created within problem-solving regarding PA plans and whether they can be categorized using existing taxonomies or classification systems. Almost two-thirds (3393/5252, 64.6%) of coping strategies could be coded using classes from the compendium of self-enactable techniques. The added coping strategies were adapting the existing plan in different ways or managing one’s time and required equipment in order to avoid interference with the plan. On top of that, managing negative bodily states, such as fatigue or hunger, was identified as a relevant coping strategy. This study is the first step to classifying the content of coping strategies. This kind of classification will be necessary to provide personalized recommendations within digital planning interventions to promote PA and should be complemented by input from domain experts.

## Abbreviations

BCT: behavior change technique
OSF: Open Science Framework
PA: physical activity

## Data Availability

The data sets generated during and analyzed during this study are available in the Open Science Framework repository [36].
